# Validation of Discrete and Regression-Based Performance and Cognitive Fatigability Normative Data for the Paced Auditory Serial Addition Test in Multiple Sclerosis

**DOI:** 10.3389/fnins.2021.730817

**Published:** 2021-11-15

**Authors:** Caitlin S. Walker, Jason A. Berard, Lisa A. S. Walker

**Affiliations:** ^1^Department of Psychology, Carleton University, Ottawa, ON, Canada; ^2^The Ottawa Hospital Research Institute, Ottawa, ON, Canada; ^3^The University of Ottawa Brain and Mind Research Institute, Ottawa, ON, Canada

**Keywords:** PASAT, cognitive fatigability, fatigue, normative data, regression-based norms, discrete norms, multiple sclerosis

## Abstract

Cognitive fatigability is an objective performance decrement that occurs over time during a task requiring sustained cognitive effort. Although cognitive fatigability is a common and debilitating symptom in multiple sclerosis (MS), there is currently no standard for its quantification. The objective of this study was to validate the Paced Auditory Serial Addition Test (PASAT) discrete and regression-based normative data for quantifying performance and cognitive fatigability in an Ontario-based sample of individuals with MS. Healthy controls and individuals with MS completed the 3″ and 2″ versions of the PASAT. PASAT performance was measured with total correct, dyad, and percent dyad scores. Cognitive fatigability scores were calculated by comparing performance on the first half (or third) of the task to the last half (or third). The results revealed that the 3″ PASAT was sufficient to detect impaired performance and cognitive fatigability in individuals with MS given the increased difficulty of the 2″ version. In addition, using halves or thirds for calculating cognitive fatigability scores were equally effective methods for detecting impairment. Finally, both the discrete and regression-based norms classified a similar proportion of individuals with MS as having impaired performance and cognitive fatigability. These newly validated discrete and regression-based PASAT norms provide a new tool for clinicians to document statistically significant cognitive fatigability in their patients.

## Introduction

Cognitive impairment affects up to 70% of individuals with multiple sclerosis (MS), with impaired working memory and information processing speed abilities being fundamental cognitive deficits (Chiaravalloti and DeLuca, 2008; Grzegorski and Losy, 2017). Cognitive impairment arises in MS due to pathophysiological processes that result in lesions in the brain’s white and gray matter (DeLuca et al., 2020). Those with primary progressive (PPMS) and secondary progressive (SPMS) disease subtypes demonstrate more pronounced cognitive deficits than those with the relapsing-remitting subtype (RRMS; Eijlers et al., 2018). Furthermore, cognitive impairment tends to increase with disease duration (Amato et al., 2006).

Many individuals with MS report experiencing cognitive fatigue, or a lack of mental energy required for sustained cognitive tasks (Fisk et al., 1994). Cognitive fatigue is a debilitating symptom that can result in difficulties completing tasks at work or school that require sustained cognitive effort. Additionally, cognitive fatigue has a variety of socioeconomic consequences, such as a loss of work hours, unemployment, and early retirement (Smith and Arnett, 2005; Simmons et al., 2010). Despite these functional impairments, there is currently no universally accepted method for measuring cognitive fatigue in the MS literature.

The causes of MS-related cognitive fatigue can be classified into primary and secondary mechanisms (Forwell et al., 2008; Braley and Chervin, 2010). Secondary mechanisms include other symptoms that worsen fatigue, such as depression, mobility inefficiency, respiratory problems, and sleep disorders. The relation between depression and fatigue in MS remains unclear, with several studies reporting little to no correlation between the two symptoms even though they often overlap in MS (Krupp et al., 1988, 1989; Vercoulen et al., 1996). However, other studies have found that depression is a predictor of cognitive fatigue in individuals with MS (Berard et al., 2019b) and that there is a moderate correlation between MS-related cognitive fatigue and depression (Ford et al., 1998). It has also been postulated that reduced sleep quality due to impaired slow wave sleep may contribute to cognitive fatigue in MS (Touzet, 2017).

Primary mechanisms of cognitive fatigue, in contrast, are those that are directly related to the pathogenesis of MS, such as proinflammatory cytokines, endocrine influences, axonal loss, and an altered pattern of cerebral activation (Braley and Chervin, 2010; Linnhoff et al., 2019). In particular, CF is associated with disruptions in circuits involved in attention and arousal, including the basal ganglia, frontal cortex, and thalamus (Chaudhuri and Behan, 2000). Lesions in pathways of the reticular and limbic systems and basal ganglia being particularly implicated in CF (Chaudhuri and Behan, 2004). Functional neuroimaging studies have also demonstrated differences in activation patterns in the attention network between individuals with MS and healthy controls before, during, and after a cognitively fatiguing task (Berard et al., 2019a). In addition to structural disease pathology, pro-inflammatory cytokines have been postulated to play a role in MS-related fatigue. Individuals with MS who subjectively report higher levels of fatigue show higher levels of tumor necrosis factor alpha which was correlated with daytime sleepiness (Heesen et al., 2006).

Traditionally, MS-related cognitive fatigue has been measured subjectively through self-report questionnaires and rating scales, such as the Fatigue Severity Scale (Krupp et al., 1989), the Fatigue Impact Scale (Fisk et al., 1994), the Fatigue Scale for Motor and Cognitive Functions (Penner et al., 2009), and the Wurzburg Fatigue Inventory for MS (Flachenecker et al., 2006). Because fatigue is a multidimensional construct, however, self-report measures vary in what aspect of fatigue they measure (e.g., fatigue severity, duration, momentary perceptions, chronic character, mental or physical dimensions, and/or impact on daily functioning; Beckerman et al., 2020). In addition, self-report measures of fatigue are often prone to recall bias, regression to the mean, and they have been found to correlate weakly with one another (Fiene et al., 2018; Linnhoff et al., 2019). Prior studies have also found that there is no relationship between subjective and objective measurements of cognitive fatigue (Parmenter et al., 2003; Bryant et al., 2004; Bailey et al., 2007). Given the variability of how cognitive fatigue has been measured in the MS literature, Linnhoff et al. (2019) proposed a taxonomy that distinguishes cognitive fatigue from cognitive fatigability (CF). In contrast to cognitive fatigue that is measured subjectively, CF refers to an objectively measured decrease in performance throughout the duration of a sustained cognitive task (Walker et al., 2012).

The Paced Auditory Serial Addition Test (PASAT; Gronwall, 1977; Rao, 1990) is a sensitive measure of working memory and information processing speed that is consistently used in studies examining CF in MS. During the PASAT, participants listen to a series of single digit numbers and must add each number that is heard to the number immediately prior to it. Participants must respond orally before the presentation of the next digit to receive a correct response. The interstimulus interval (ISI) can be varied, with 3- or 2-s (3″ or 2″) ISIs being the most common in the MS literature (Tombaugh, 2006). The PASAT can measure CF when performance is compared between the beginning and end of the task, with decreased performance at the end compared to the beginning of the task being an indication of CF. Prior studies have demonstrated CF in MS by comparing performance between the first and second half of the task (Walker et al., 2012; Berard et al., 2014) and the first and last third of the task (Morrow et al., 2015; Berard et al., 2018, 2019b). In both cases, individuals with MS have demonstrated greater within-task performance decrements compared to healthy controls. Agyemang et al. (2021) investigated how performance declines over time on the PASAT with the same population used in the current study. They found that individuals with MS had more cumulative errors throughout the task than the healthy controls, particularly for the 3″ PASAT. When compared to controls, the MS group had a steeper, linear performance decline from the start of the task rather than their performance breaking down at any specific point during the task. Therefore, the CF experienced by individuals with MS seems to arise from difficulties maintaining an optimal level of performance from the initial onset of a cognitively demanding task.

PASAT scores typically constitute the total number of correct responses for each trial, out of a maximum score of 60. A disadvantage of using total correct scoring is that participants may use a chunking strategy to reduce the working memory demands of the task. Namely, some participants may add two numbers, skip the next number, then add the following two numbers which reduces both the difficulty of the task and its sensitivity at detecting cognitive impairment (Snyder et al., 1993). Dyad and percent dyad scoring methods can be used to better determine whether a participant was performing the task as intended. Total dyad scores are calculated by summing the *number of times* two correct responses occur in succession, while percent dyad scores reflect the *proportion of time* that two correct responses occurred in a row. Thus, dyad and percent dyad scores reflect whether, and for what proportion of time, the participant was meeting the working memory demands of the task.

To evaluate an individual’s performance on a neuropsychological test relative to demographically similar healthy individuals, clinicians consult normative data. Discrete norms are derived by dividing data into groups with certain demographic variables (e.g., age, sex, and education brackets) and computing the mean and standard deviation (SD) for each group. Typically, performance is considered impaired if an individual scores 1.5 SD or more below the normative mean. However, there are limitations to using discrete norms, including arbitrary cut-offs for age and education grouping that can affect the interpretation of an individual’s impairment depending on which category they are assigned to Oosterhuis et al. (2016). Other limitations include a small number of individuals in each grouping and a lack of correction for all relevant demographic information (Berrigan et al., 2014). Regression-based norms can be used to overcome these limitations. They are derived by computing linear regressions that control for numerous demographic variables (Testa et al., 2009; Bergman and Almkvist, 2015). Moreover, smaller sample sizes can be used to obtain norms as precise as those obtained from discrete norms (Oosterhuis et al., 2016).

Berard and Walker (2021) established discrete and regression-based normative data for PASAT performance and CF using data from 178 healthy control participants. They established regression-based formulae that were demographically adjusted for sex, age, and number of years of education. Additionally, discrete normative data were established by subdividing participants by number of years of education (≤15 years or ≥ 16 years) and age (20–35, 36–50, and 51–65 years of age). Because no significant differences were found between males and females on PASAT performance and CF, the discrete norms were not divided by sex. For both performance and CF, norms were computed for the entire task, for each half of the task, and for each third of the task for the 3″ and 2″ versions.

Although CF is known to be a debilitating symptom for individuals with MS, it was unknown how much CF was experienced by a healthy population. To date, there has not been a universally accepted standard for quantifying a normal amount of CF. Therefore, the goal of the normative data, as previously established by Berard and Walker (2021), was to establish how much CF was experienced by healthy control participants. This data was validated in the current study to determine how well the previous normative data could classify individuals with MS as having impaired performance and CF.

The first objective of the current study was to validate the PASAT discrete and regression-based normative data for quantifying performance and CF in an Ontario sample of individuals with clinically definite MS. The second objective of this study was to determine whether the discrete or regression-based norms were more sensitive to impaired performance and CF in individuals with MS. It was hypothesized that individuals with MS would perform worse than healthy controls on PASAT performance and CF measures. Secondly, it was expected that regression-based norms would classify a greater number of individuals with MS as impaired on PASAT performance and CF than the discrete norms.

## Materials and Methods

### Participants

Participants consisted of 178 healthy controls previously used to establish normative data (Berard and Walker, 2021) and 186 individuals with a confirmed diagnosis of MS. Participants with MS were recruited from the Ottawa Hospital MS Clinic. They were informed about the research study by their treating team and those who indicated interest were then contacted by research staff. Healthy control participants were recruited from the community through word of mouth, posted advertisements, and newspaper and website advertisements. Inclusion criteria for all participants included being between 18 and 65 years of age and fluency in English. A confirmed diagnosis of MS was also required for the MS group for inclusion in the study. Exclusion criteria for all participants included any neurological, medical, or psychiatric condition (besides MS and depression) that might impede cognition, use of legal or illegal drugs that might impact cognition, prior head trauma, a learning disability, attention-deficit hyperactivity disorder, mild cognitive impairment (aside from that related to MS), dementia, or substance abuse.

### Procedure and Measures

All participants volunteered to participate in one of three separate studies evaluating cognition in individuals with MS. All three studies contributing to the current project were approved by the Ottawa Health Science Network Research Ethics Board with one of the studies also being approved by the Sunnybrook. Prior to test administration, study procedures were reviewed with all participants, and they were given the opportunity to ask questions. Thereafter, all participants provided their full informed consent. Participants completed a comprehensive battery of neuropsychological tests, including the PASAT, that evaluated multiple cognitive domains. The PASAT was administered as either the third or fourth task in the battery in each of the three studies, such that relative time of administration was unlikely to be a significant factor in performance.

The PASAT version used in the Multiple Sclerosis Functional Composite (MSFC; Cutter et al., 1999) was utilized, with the 3″ PASAT being administered before the 2″ PASAT. Each test consisted of 60 trials. Research assistants trained by a licensed Clinical Neuropsychologist recorded oral PASAT responses at both the 3″ and 2″ ISIs. The total number of correct responses, dyad scores, and percent dyad scores were recorded. CF was measured by creating difference scores between the second and first half of the task as well as the last third and first third of the task. Halves were derived by subtracting the score of the first 30 trials from the second 30 trials (i.e., second half score—first half score). Thirds were derived by subtracting the score of the first 20 trials from the last 20 trials (i.e., last third score—first third score). In order to have an equal number of possible dyads in each portion of the task, a correct dyad was scored for a correct response on the first pair of numbers presented (Fisk and Archibald, 2001). Percent dyad scores were calculated using the following formula: (Dyad Score/Total Correct Score) × 100%. Z-scores for PASAT performance and CF were computed using the discrete norms and regression-based formulae established by Berard and Walker (2021). Participants were classified as impaired in their z-scores were ≥ 1.5 SD below the normative mean.

### Analyses

First, analyses were conducted to determine if there were differences between the MS and healthy control group in sex, age, and number of years of education. A chi-square test for independence was used to examine group differences in the proportion of males and females and one-way analyses of variance (ANOVAs) compared group differences in age and number of years of education. A series of one-way analysis of covariance (ANCOVA) tests examined group differences in raw scores of the performance and CF measures of the 3″ and 2″ PASAT. Because there was a group difference in number of years of education, it was included as a covariate for all ANCOVAs. A one-way ANOVA was then performed to determine whether performance and CF scores for the MS group differed between the three studies from which the data for the current study were derived. Then, for the MS group, the proportion of performance and CF z-scores from the discrete and regression-based norms that were ≥ 1.5 SD below the normative mean were computed. Finally, chi-square tests for independence were used to test whether discrete or regression-based norms classified a greater number of individuals with MS as impaired on performance and CF.

## Results

### Demographics and Disease Characteristics

Information on the demographics and disease characteristics for the MS and healthy control groups is shown in Table 1. In the MS group, there was a high proportion of those with RRMS (84.4%) compared to SPMS (11.8%) and PPMS (3.8%). The MS group also had a mean disease duration of 7.2 years and a mean Expanded Disability Status Scale (EDSS) score of 2.3, indicating that participants had minimal to mild disability on average (Kurtzke, 1983). There were no statistically significant group differences in sex or age. Given that education was different between groups, it was thereafter included as a covariate in subsequent analyses.

**TABLE 1:** Demographic information and disease characteristics.

Demographic variable	Controls	MS	χ^2^	*F*	*p*
Sex. *n* (%)	*M* = 34 (19.1) *F* = 144 (80.9)	*M* = 41 (22) *F* = 145 (78)	0.48		0.488
Age (years), *M* (*SD*)	41.5 (12.1)	43.1 (9.7)		1.95	0.163
Education (years), *M* (*SD*)	15.8 (2.4)	15.1 (2.2)		8.64	0.004**
EDSS, *M* (*SD*)		2.3 (1.5)			
Disease duration (years), *M* (*SD*)		7.2 (5.4)			
MS Subtype, *n* (%)		RRMS = 157 (84.4) SPMS = 22 (11.8) PPMS = 7 (3.8)			

M, males; F, females; EDSS, Expanded Disability Status Scale score; RRMS, relapsing remitting multiple sclerosis; SPMS, secondary progressive multiple sclerosis; PPMS, primary progressive multiple sclerosis. **p < 0.01.

### Study Differences

Given that the sample was comprised of individuals from three different contributing studies, potential differences between studies in PASAT performance and CF were investigated. Significant between-study differences in PASAT performance measures are shown in Table 2 for the MS group. There were statistically significant between-study differences for total correct and dyad performance measures of the 2″ PASAT. Significant between-study differences in PASAT CF measures for the MS group are shown in Table 3. There were statistically significant differences between studies for percent dyad CF scores on the 2″ PASAT for both halves and thirds. There were no statistically significant study differences for the 3″ PASAT.

**TABLE 2:** Between-study differences in 2″ PASAT performance measures for the MS group.

Measure	IPSIMS *M* (*SD*)	BICAMS *M* (*SD*)	SUNSCREEN *M* (*SD*)	*F*	*p*
Total correct	19.9 (12.0)	31.7 (12.7)	29.5 (11.0)	17.93	<0.001***
Dyad	11.0 (10.3)	20.3 (14.8)	17.4 (12.8)	9.13	<0.001***

*IPSIMS, Information processing speed in MS study; BICAMS, Brief international cognitive assessment for MS study; SUNSCREEN, Using computers to assess cognition in MS study.* ***p < 0.001.

**TABLE 3:** Between-study differences in 2″ PASAT cognitive fatigability measures for the MS group.

Measure	IPSIMS *M* (*SD*)	BICAMS *M* (*SD*)	SUNSCREEN *M* (*SD*)	*F*	*p*
Percent dyad (Halves)	−30.4 (28.4)	−17.6 (17.6)	−16.0 (20.8)	7.38	0.001**
Percent dyad (Thirds)	−35.7 (29.6)	−22.7 (21.1)	−24.3 (21.3)	5.22	0.006**

PSIMS, Information processing speed in MS study; BICAMS, Brief international cognitive assessment for MS study; SUNSCREEN, Using computers to assess cognition in MS study. **p < 0.01.

### Group Differences

#### Performance

**FIGURE 1:**
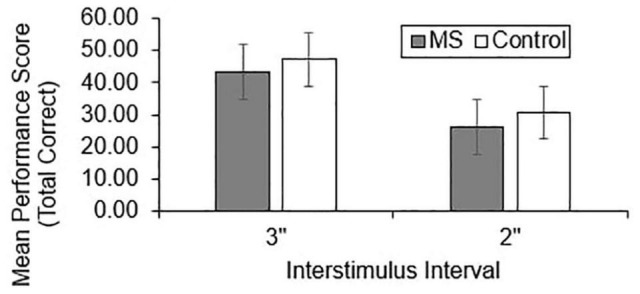
Group difference in total correct performance scores.

**FIGURE 2:**
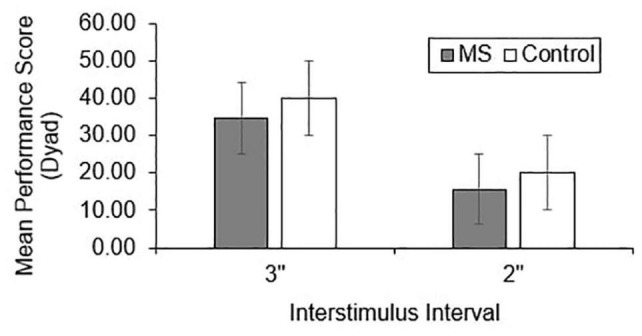
Group difference in dyad performance scores.

**FIGURE 3:**
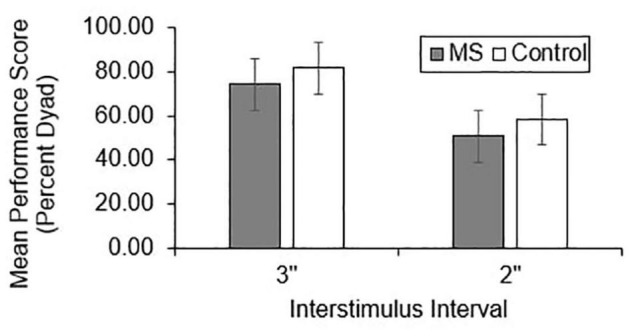
Group difference in percent dyad performance scores.

Group differences in total correct (Figure 1), dyad (Figure 2), and percent dyad (Figure 3) scores were examined for overall PASAT performance. There were statistically significant group differences in 3″ PASAT performance using total correct scoring [*F*_(1, 358)_ = 10.34, *p* = 0.004], dyad scoring [*F*_(1, 358)_ = 9.38, *p* = 0.002], and percent dyad scoring [*F*_(1, 358)_ = 9.05, *p* = 0.003]. There were also statistically significant group differences using total correct scoring [*F*_(1, 351)_ = 5.84, *p* = 0.016], dyad scoring [*F*_(1, 351)_ = 5.92, *p* = 0.015], and percent dyad scoring [*F*_(1, 351)_ = 5.94, *p* = 0.015] for the 2″ PASAT. The MS group scored lower on all performance measures than the healthy control group.

#### Cognitive Fatigability

**FIGURE 4:**
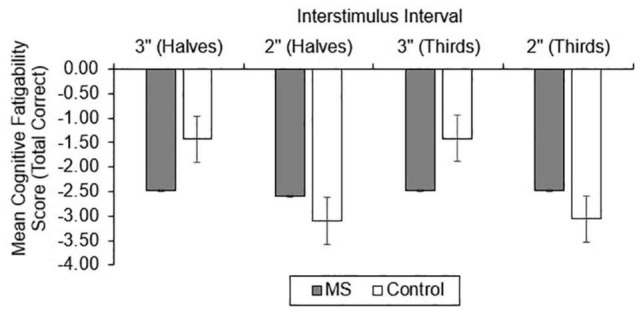
Group difference in total correct cognitive fatigability scores.

**FIGURE 5:**
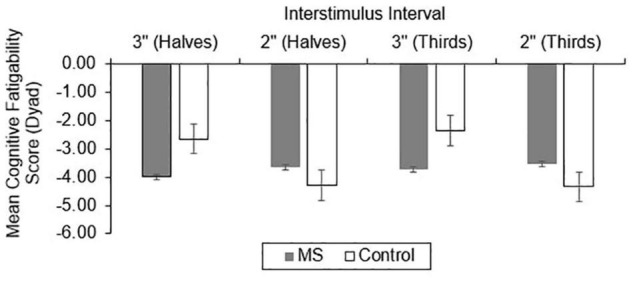
Group difference in dyad cognitive fatigability scores.

**FIGURE 6:**
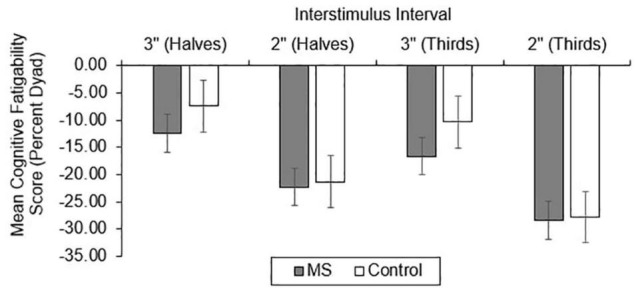
Group difference in percent dyad cognitive fatigability scores.

For CF, group differences in total correct (Figure 4), dyad (Figure 5), and percent dyad (Figure 6) scores were also examined. Individuals with MS performed significantly worse than healthy controls on all CF measures for the 3″ PASAT. Using halves, the two groups significantly differed in total correct scores [*F*_(1, 358)_ = 6.57, *p* = 0.011], dyad scores [*F*_(1, 358)_ = 4.85, *p* = 0.028], and percent dyad scores [*F*_(1, 358)_ = 6.45, *p* = 0.012]. Using thirds, the two groups also significantly differed in total correct scores [*F*_(1, 358)_ = 8.50, *p* = 0.004], dyad scores [*F*_(1, 358)_ = 6.87, *p* = 0.009], and percent dyad scores [*F*_(1, 358)_ = 7.54, *p* = 0.006].

There were no statistically significant group differences in CF for the 2″ PASAT when halves were used to calculate total correct scores [*F*_(1, 350)_ = 1.01, *p* = 0.316], dyad scores [*F*_(1, 350)_ = 1.46, *p* = 0.228], or percent dyad scores [*F*_(1, 350)_ = 0.00, *p* = 0.959]. In addition, there were no statistically significant group differences in CF on the 2″ PASAT when thirds were used to calculate total scores [*F*_(1, 350)_ = 1.74, *p* = 0.189], dyad scores [*F*_(1, 350)_ = 3.08, *p* = 0.080], or percent dyad scores [*F*_(1, 350)_ = 0.02, *p* = 0.898]. Thus, the MS group performed significantly worse than healthy controls on all CF measures only when a 3″ ISI was used.

### Impairment

The proportion of individuals with MS who demonstrated impaired performance (Table 4) and CF (Table 5) using the discrete and regression-based norms was determined for all scoring methods. In contrast to the hypothesis that regression-based norms would be more sensitive to impaired performance and CF than the discrete norms, there were no statistically significant differences in the number of individuals with MS who were classified as impaired.

**TABLE 4:** The proportion of the MS group impaired on PASAT performance measures.

Measure	Discrete norms,% impaired	Regression-based norms,% impaired
**3″ PASAT**		
Total correct	18.3	18.3
Dyad	19.9	21.5
Percent dyad	18.8	16.7
**2″ PASAT**		
Total correct	13.4	14.5
Dyad	1.6	4.8
Percent dyad	15.1	14.0

**TABLE 5:** The proportion of the MS group impaired on PASAT cognitive fatigability measures.

Measure	Discrete norms, % impaired	Regression-based norms, % impaired
**3″ PASAT (Halves)**		
Total correct	12.9	12.9
Dyad	8.6	9.7
Percent dyad	14.5	13.4
**2″ PASAT (Halves)**		
Total correct	5.9	4.8
Dyad	7	5.4
Percent dyad	9.1	7
**3″ PASAT (Thirds)**		
Total correct	12.4	9.1
Dyad	10.2	9.7
Percent dyad	15.1	12.9
**2″ PASAT (Thirds)**		
Total correct	4.3	4.3
Dyad	4.3	4.3
Percent dyad	10.2	8.1

## Discussion

CF is a common and challenging symptom for individuals with MS that negatively affects daily functioning, increases the likelihood of unemployment, and reduces quality of life. Although CF is known to be a debilitating symptom for individuals with MS, there is currently no universally accepted method for quantifying it. Therefore, the goal of this study was to validate discrete and regression-based normative data established by Berard and Walker (2021) to detect impaired performance and CF in an Ontario sample of individuals with MS. The second objective of this study was to determine whether discrete or regression-based norms were more sensitive to impaired performance and CF in individuals with MS. The results validated both the discrete and regression-based PASAT norms for use with individuals with MS. However, there was no advantage of using regression-based norms to detect impaired performance or CF compared to using discrete norms.

### Group Differences

#### Performance

Individuals with MS demonstrated significantly lower raw PASAT scores than healthy controls regardless of the scoring method or ISI that was used. These results are consistent with previous studies that have demonstrated worse PASAT performance in individuals with MS compared to healthy controls using total correct (Kujala et al., 1995; Rosti et al., 2006; Solari et al., 2007), dyad (Kujala et al., 1995; Snyder and Cappelleri, 2001; Snyder et al., 2001; Rosti et al., 2006; Solari et al., 2007), and percent dyad scoring methods (Fisk and Archibald, 2001; Rosti et al., 2006). Thus, the performance of an Ontario-based early sample is in keeping with what has been documented in other studies.

#### Cognitive Fatigability

Consistent with our expectations, the results showed that individuals with MS demonstrated greater CF than healthy controls using all scoring methods on the 3″ PASAT. This was true whether halves or thirds were used, suggesting that both methods detected significantly more CF in the MS group than the healthy control group. However, there were no significant group differences in CF for the 2″ PASAT when either halves or thirds were used. This might be explained by the increased difficulty of the 2″ PASAT which likely challenged the cognitive capacity of both groups from the onset of the task. As a result, CF scores did not differ between the two groups. Because the 2″ PASAT was always administered after the 3″ version, another possibility is that participants were already fatigued by the time they completed the 2″ version. Given the effort required to maintain performance at the prior 3″ ISI, it may have resulted in impaired performance from the onset of the 2″ task.

### Impairment

#### Performance

Across all scoring methods, both the discrete and regression-based norms classified a greater proportion of individuals with MS as impaired using the 3″ compared to the 2″ PASAT. As previously discussed, a shorter ISI likely increased the difficulty of the PASAT even for the healthy control participants, resulting in decreased sensitivity at detecting impaired performance in individuals with MS, given the difficulties experienced by both groups. For the 3″ PASAT, all scoring methods, for both types of norms, classified a similar proportion of participants (all over 16%) as having impaired PASAT performance (Table 4). For the 2″ PASAT, dyad scoring classified a much smaller proportion of individuals with MS as impaired (1.6–4.8%) compared to total correct and percent dyad scoring (13.4–15.1%; Table 4). This lower proportion of impairment is expected given the increased difficulty of the task and the fact that participants are less likely to obtain two correct scores in a row. This again justifies the use of the 3″ PASAT in the MSFC over the 2″ version given its greater sensitivity to impairment.

#### Cognitive Fatigability

Similar to the performance impairments, both the discrete and regression-based norms classified a greater proportion of individuals with MS as having impaired CF using the 3″ compared to the 2″ PASAT. This suggests that the 2″ PASAT was difficult and fatiguing for both groups and likely resulted in floor effects. The 2″ PASAT may be too difficult for participants from the beginning of the task thereby making it less likely to detect a breakdown in performance over the course of the task (i.e., as many errors occur at the beginning of the task as at the end of the task). However, for both ISIs, halves and thirds were equally sensitive to impaired CF. This suggests that comparing scores between the first half and last half of the PASAT, or the first and last third of the PASAT, are both effective methods for detecting impaired CF in MS. Additionally, for both ISIs and norm types, percent dyad scoring classified the largest proportion of individuals with MS as having impaired CF compared to total correct and dyad scoring methods.

The results of the current study support prior research by Walker et al. (2012) who found that percent dyad scoring was most sensitive to CF for the 3″ PASAT, while both groups had difficulties meeting task demands for the 2″ PASAT. Thus, regardless of whether one is interested in detecting group differences or impairment in performance and CF, a decrease over time in the proportion of time spent appropriately meeting the working memory demands of the PASAT appears to be the most sensitive manner of CF detection. As the task progresses, those with MS are less able than healthy controls to meet the working memory demands of the task and also demonstrate the highest rate of impairment when this scoring method is used for the 3″ PASAT (12.9–15.1%; Table 5).

### Discrete and Regression-Based Norms

In contrast to our hypothesis, there were no statistically significant differences in the proportion of individuals with MS who were classified as having impaired performance or CF using the regression-based norms compared to the discrete norms. Therefore, the addition of sex in the regression-based formulae (over and above the age and number of years of education accounted for in the discrete norms) did not improve the sensitivity at detecting impairment for either performance or CF in the MS group. This is in line with previous research demonstrating that performance on the PASAT is not affected by sex or gender (Johnson et al., 1988; Roman et al., 1991; Wingenfeld et al., 1999; Fluck et al., 2001) or that the effect of sex was very small and not clinically significant (Brittain et al., 1991; Wiens et al., 1997; Diehr et al., 1998).

That regression-based norms did not detect higher rates of impairment was unexpected given that prior research has shown that regression-based norms are typically more sensitive than discrete norms for capturing cognitive functioning in MS (Smerbeck et al., 2012; Burggraaff et al., 2017). However, the lack of similar findings in the current study might be explained by the fact that additional variables, such as ethnicity, were not accounted for. Age, education, and ethnicity have been found to be significant predictors of PASAT performance in prior studies (Diehr et al., 1998, 2003). Discrepancies in neuropsychological test performance between different ethnicities have been explained by socioeconomic status, which has been found to correlate highly with neuropsychological test scores and the risk of disability from MS (Gasquoine, 2009; Calocer et al., 2020). Separate from the number of years of education, there have also been historical differences in the quality of education afforded to different ethnicities (Manly et al., 2002; O’Bryant et al., 2007) which might impact neuropsychological test performance. Thus, ethnicity is an important variable to control for in future PASAT normative data.

### Limitations and Future Directions

The present study was the first to validate PASAT discrete and regression-based normative data for measuring CF in an early Ontario sample of individuals with MS. Despite the important implications of the results, the study is not without limitations. One limitation is that the data were analyzed retrospectively and were derived from three prior studies. Measures that were used in the neuropsychological evaluations varied between the three studies, resulting in the inability to examine potential correlations between CF and subjective fatigue or depression. In addition, medication use was not examined. It is possible that some participants were taking medications that improved their fatigue, such as fampridine-SR and/or antidepressants. Therefore, future research should examine medication use or exclude participants taking medications that might impact their CF results.

Another limitation of this study was the characteristics of the sample. Because the sample was exclusively from Ontario, it is unclear how the impairment classification rates would differ in other regions given potential differences in demographics. Additionally, the majority of participants in the study had RRMS, a low disease duration, and low levels of disability, reflected by a low mean EDSS score. These characteristics might have impacted the type and extent of cognitive deficits that were observed since individuals with RRMS typically demonstrate milder information processing speed deficits than those with progressive disease subtypes (De Sonneville et al., 2002; Benedict et al., 2006; Potagas et al., 2008). Furthermore, individuals with MS with shorter disease durations tend to show more subtle cognitive impairments than those with longer disease durations (Amato et al., 1995, 2001; Achiron et al., 2013). The fact that rates of CF impairment up to 15% were detected in this sample despite the low disease duration and level of disability speaks to the sensitivity of the norms at detecting even subtle cognitive changes early in the disease course. CF is correlated with MS pathophysiology (Manjaly et al., 2019), with the pathophysiology being more pronounced in progressive subtypes compared to RRMS (Dutta and Trapp, 2014). Future work should, therefore, validate the PASAT normative data for performance and CF in a sample with a longer disease duration and in more individuals with progressive disease subtypes since this sample may be more likely to show evidence of CF.

Another direction for future research is to develop CF norms for other neuropsychological tests of information processing speed and working memory and to validate these norms in a sample of individuals with MS. In prior studies, CF has been investigated using the Blocked Cyclic Naming Task (Cehelyk et al., 2019), the Stroop task (Chinnadurai et al., 2016), the Symbol Digit Modalities Test (DeLuca et al., 2008; Chinnadurai et al., 2016), and the *n*-back task (Bailey et al., 2007). In particular, future research should aim to establish concurrent validity with these measures.

Future research should also aim to establish effective interventions to target CF. There is currently a lack of clear recommendations on how CF should be measured and treated (Walker et al., 2019). Past treatments have included a pharmacological intervention using fampridine-SR (Morrow et al., 2017) and a procedural intervention using transcranial direct current stimulation (Fiene et al., 2018), with only the procedural intervention demonstrating efficacy in treating CF. There is a need to investigate whether or not behavioral interventions are efficacious. Given that such interventions have been successful in targeting subjective fatigue (Plow et al., 2019), it would be prudent to see if modifications of such techniques could prove beneficial for CF as well.

## Conclusion

In conclusion, the current study validated the PASAT discrete and regression-based norms for performance and CF in a sample of individuals with MS. These results have important implications, given that the inability to maintain optimal cognitive performance over a long period of time may limit an individual’s productivity and ability to concentrate at work or school. For clinicians, these results provide a new tool for documenting statistically significant CF in their patients. Overall, the results revealed that the 3″ PASAT is sufficient for detecting impaired performance and CF. Scoring CF using halves and thirds were found to be equally effective methods for detecting impairment. Finally, both the discrete and regression-based norms were equally effective at detecting impaired performance and CF in this sample.

## Data Availability Statement

The datasets presented in this article are not readily available because participants of this study did not consent for their data to be shared publicly. Requests to access the datasets should be directed to corresponding author.

## Ethics Statement

The studies involving human participants were reviewed and approved by the Ottawa Health Science Network Research Ethics Board. The patients/participants provided their written informed consent to participate in this study.

## Author Contributions

CW contributed to the design of the study, conducted analyses, and wrote and edited the article. JB conceived and designed the study, conducted analyses, and edited the article. LW conceived and designed the study as well as edited the article. All authors contributed to the article and approved the submitted version.

## Conflict of Interest

The authors declare that the research was conducted in the absence of any commercial or financial relationships that could be construed as a potential conflict of interest.

## Publisher’s Note

All claims expressed in this article are solely those of the authors and do not necessarily represent those of their affiliated organizations, or those of the publisher, the editors and the reviewers. Any product that may be evaluated in this article, or claim that may be made by its manufacturer, is not guaranteed or endorsed by the publisher.
